# Understanding Drivers of Nurse Migration and Its Consequences Amid the Polycrisis in North Lebanon

**DOI:** 10.7759/cureus.91676

**Published:** 2025-09-05

**Authors:** Maha J Dankar, Walid Ammar

**Affiliations:** 1 Health Policy, Higher Institute of Public Health, Faculty of Medicine, Saint Joseph University of Beirut, Beirut, LBN

**Keywords:** healthcare workforce, health policy, health workers well-being, lebanese crisis, nurses migration, qualitative research

## Abstract

Background and objective: Lebanon's healthcare system has faced immense pressure in recent times due to a multifaceted crisis, resulting in widespread nurse migration and worsening the existing chronic shortage. This study aims to explore nurse migration in the underserved area of North Lebanon, focusing on its drivers, perceived impact on care quality, and the coping strategies of remaining nurses.

Methods: A qualitative design was adopted, using semi-structured interviews with 24 nurses currently working in hospitals across North Lebanon. Deductive and inductive techniques were used to analyze the collected data.

Results: Economic hardship, worsened by Lebanon’s currency devaluation, emerged as the leading driver of nurse migration, followed by poor working conditions and job opportunities abroad. The loss of experienced staff led to a decline in care quality and an increased workload for remaining nurses. Psychological and emotional strain was common, with coping strategies including income diversification, personal resilience, and support from family and peers. Notably, the crisis has reversed the role of family obligations from a retention factor to a push factor and highlighted the growing influence of peer networks in migration decisions.

Conclusion: Economic instability and inadequate compensation were key factors driving nurse migration, particularly in underserved areas. The findings underscore the urgent need for systemic reforms to reduce workloads and prevent further deterioration in healthcare services.

## Introduction

The global nursing shortage poses a significant threat to healthcare systems [1], with an anticipated shortfall of 13 million nurses worldwide by 2030, especially affecting low- and lower-middle-income countries (LMICs) [2]. In the Eastern Mediterranean Region, the World Health Organization (2020) has identified critical understaffing, which has further burdened already fragile health systems [3].

Lebanon has experienced a series of crises since late 2019, resulting in a 40% contraction in gross domestic product (GDP), a 98% currency depreciation, and soaring inflation [4]. These conditions have compelled skilled health workers, including nurses, to emigrate [5]. According to the World Health Organization (2020), Lebanon had a nurse-to-population ratio of just 1.67 per 1,000, far below the global average of 3.7, reflecting the healthcare sector being severely strained, even before the full impact of the crisis unfolded [6]. Nurse migration has since intensified workloads [7], leading to burnout, absenteeism, and a decline in care quality [5,8-10].

Global evidence suggests that economic crises worsen healthcare worker migration [11,12]. In Lebanon, this trend is compounded by inadequate support systems and limited funding [9,13]. Despite the severity of the situation, limited research exists examining the effects of Lebanon’s compounded crises on nurse migration, particularly in underserved regions.

This study seeks to address this gap by examining the dynamics of nurse migration in North Lebanon. Specifically, it aims to explore the driving factors of nurse migration in hospitals in North Lebanon, assess the perceived impact of nurse migration on the quality of care, and identify the coping strategies adopted by the nurses who remain in these hospitals.

## Materials and methods

Study design 

This study employed a qualitative research design to examine the causes and consequences of nurse migration during multiple crises. Semi-structured interviews were conducted to explore participants’ reasons for migration, the perceived impact on quality of care, and the coping strategies adopted by remaining nurses. The study was undertaken in hospitals located in North Lebanon, considered as underserved areas [14]. A total of 12 hospitals (nine private and three public) were selected based on pre-crisis and post-crisis operational bed numbers (more than 55 operational beds).

Of the 21 private hospitals in North Lebanon, six were excluded due to inactivity or insufficient bed capacity, and six declined to participate. The nine private hospitals included in the study represent over 60% of the region’s private hospital bed capacity. Among public hospitals, four were excluded for similar reasons, and two refused to participate. The remaining three public hospitals account for over 50% of public hospital beds in the area.

Sample

A total of 24 nurses were purposively selected across 12 hospitals, with two participants recruited per site. Selection was guided by professional role, years of experience, and availability, ensuring variation across eight nursing supervisors (NSs), 11 registered nurses (RNs), and five practical nurses (PNs). Hospital managers provided lists of eligible staff, but they were not involved in contacting participants directly. The selection of two nurses per site was strategically chosen to capture diverse perspectives across hospitals while maintaining a manageable sample for in-depth qualitative interviews.

Data collection 

Approvals were obtained from the administrations of the targeted hospitals prior to data collection. In-person interviews were scheduled according to participants’ preferences and availability. The first author conducted face-to-face interviews guided by an interview script with three open-ended questions designed for this study. Interviews were audio-recorded, and field notes were taken simultaneously to capture nonverbal cues and other contextual details. All interviews were conducted in a private room to ensure a comfortable and disturbance-free environment.

Data collection took place from early June to the end of July 2023. Each participant was allocated a code to ensure confidentiality. Interviews, conducted in Arabic, lasted between 20 and 30 minutes. This duration was not predetermined through a pilot phase but was guided by the richness of participants’ responses; in practice, it was sufficient to capture in-depth perspectives while minimizing participant fatigue. The recordings were transcribed verbatim and translated into English by a translator with medical terminology expertise. A bilingual specialist reviewed the translations, and any discrepancies were resolved, sometimes with input from the original interviewees.

The interview guide consisted of three open-ended questions exploring the reasons for migration, its impact on the quality of care, and the coping strategies adopted by remaining nurses. Each interview started with broad questions, then moved to more focused ones, with the researcher probing for clarification and deeper insight. The key questions asked were: “As a Lebanese nurse, how do you perceive the phenomenon of nurse migration? What are the main factors influencing others' decision to leave?" "In your experience, how has nurse migration affected the quality of care provided in your institution or in Lebanon more broadly?" "What coping strategies have you personally used to deal with the challenges of the migration crisis, and which ones have been most effective for you?"

Data analysis

For qualitative data analysis, a content analysis approach was applied to guide the process [15]. Data were analyzed using both deductive and inductive techniques. The deductive approach was used initially, starting with predefined questions to gather specific insights. This was followed by an inductive approach, identifying patterns and emerging categories from participants' responses [16]. This analysis focused on the development of descriptive categories relevant to each research question. A line-by-line coding process was employed, and related codes were gathered under one category.

Ethical consideration

All study protocols were approved by the Research Ethics Committee of Saint Joseph University of Beirut (approval no. USJ-2023-103), with permissions also granted by hospital administrations prior to data collection. Participants received a clear explanation of the study’s purpose and risks and benefits, and written informed consent was obtained before each interview. They were informed of their right to withdraw at any time without consequence, that participation was voluntary, and that no financial compensation would be provided. No personal identifying information was collected. All data were stored on a password-protected computer owned by the first author, with access restricted to the research team.

## Results

Characteristics of the respondents

The sample of the study comprised 24 nurses, including 17 females (70.8%) and seven males (29.2%), with a balanced range of educational and professional backgrounds. A majority (58.3%) held university-level nursing degrees (Master of Science in Nursing (MSN) and Bachelor of Science in Nursing (BSN)), while 41.7% were graduates with technical degrees (License Technique (LT), Technique Superior (TS), and Baccalaureate Technique (BT)). In terms of experience, nearly half of the participants (45.8%) had 11 to 20 years of service, followed by 33.3% with less than 10 years and 20.8% with over two decades of experience (Table 1).

**Table 1:** Demographic characteristics of study participants (n=24)

Characteristics		Category	N (%)
Gender		Male	7 (29.2%)
		Female	17 (70.8%)
Educational background	University level	Master of Science in Nursing (MSN)	6 (25.0%)
		Bachelor of Science in Nursing (BSN)	8 (33.3%)
	Institute level	License Technique (LT)	3 (12.5%)
		Technique Superior (TS)	2 (8.3%)
		Baccalaureate Technique (BT)	5 (20.8%)
Years of experience		10 years or less	8 (33.3%)
		11-20 years	11 (45.8%)
		21 years and more	5 (20.8%)

Qualitative findings

The analysis revealed three main categories: migration drivers, migration impact on the quality of care, and coping strategies adopted by the remaining nurses. The following sections present the findings for each category, structured according to deductive and inductive insights.

Migration Drivers

These included push factors and pull factors. The main push factors comprised economic hardship, unfavorable working conditions, country instability and insecurity, and psychological strain combined with familial obligations. Economic hardship emerged as the most pressing driver. The ongoing economic crisis, marked by currency devaluation and inflation, rendered salaries insufficient to meet basic needs. As RN-10 stated, “low salaries were no longer sufficient to cover necessities, including education" for their (those who migrated) children and healthcare; RN-2 added, “The salaries are not sufficient even for transportation,” while NS-4 expressed, “The financial situation is what prompted everyone to leave… Low salaries under current pressures make it impossible to afford proper education for my children.” Similar concerns were voiced by RN-10, PN-1, and PN22.

Unfavorable working conditions, such as long shifts, high workload, and persistent staff shortages, pushed nurses to migrate. A sentiment echoed by RN-11: “Long working hours, with no fixed schedule, and no vacations, to finally have a very low payment is unfair.” Job insecurity further intensified these pressures, as NS-3 noted, “We are always exposed to expulsion; especially after the end of the COVID crisis, many employees have been laid off,” while RN-5 expressed, “We as employees are not stable nor secure; we can be dismissed for no reason, as many nurses have been dismissed.” This persistent fear was echoed by other participants, including PN-1, PN-3, and RN-11.

Country instability and insecurity were also prominent drivers. It was observed by NS-1 that "The nation is unsafe in every way, politically, socially, and economically." Moreover, RN-1 emphasized the impact of personal freedom, stating that “The whole country is no longer safe; we are unable to leave homes after nine o'clock.” This widespread sense of insecurity was shared by PN-4 and RN-7.

Lastly, psychological strain combined with family obligations contributed significantly to the desire to migrate. Emotional distress was frequently cited, with RN-2 admitting, "My psychological problems increased...now I am depressed," and RN-4 stating simply, "I need a change." Nurses also felt compelled to provide for their families amid deteriorating conditions: RN-6 explained, "I want to migrate to secure my family," and PN-1 emphasized, "My only goal is my family, despite my fatigue." Adding to this, NS-4 expressed, "The weight of my obligations towards my family and the constant struggle to secure their livelihood is a significant source of increasing stress and tension." 

Pull factors encouraging nurse migration involved the availability of job opportunities abroad, promises of better salaries and work environments, and the influence of their peers who had already migrated. Availability of job opportunities was key. Many nurses were drawn to positions abroad that aligned with their qualifications and expectations. As noted by NS-6, "Offers increased, especially from Arab countries with high salaries for a large number of nurses." This perception was shared by RN-2's observation that "People have greater ambitions for the job opportunities that exist abroad," while RN-9 highlighted the local gap, noting, "Young people living in Lebanon lack job opportunities easily found in other countries." 

Promises of better salaries and work environments were another pull factor. Financial incentives and the prospect of improved work conditions were major motivators, particularly for younger and unmarried nurses. As PN-5 explained, "Many nurses were forced to travel or leave their work seeking access to improved working conditions." In addition, NS-4 stated, “The nurses who traveled have received better salaries,” and RN-3 noted, “Most nurses have left for hospitals that gave them promises and contracts of better salaries.” Beyond financial aspects, nurses also sought environments where they felt respected and valued, as PN-2 expressed, "In addition to the salary we all need, colleagues have left searching for respect, an acceptable work schedule, promotions, and a real team to work with." 

Peer influence effects were yet another pull factor. The experience of colleagues who migrated played a substantial role in influencing others to follow. In fact, RN-8 echoed this, explaining that nurses abroad are "happy and encourage the remaining nurses to leave, as they have psychological and salary comfort." Similarly, RN-5 added that those who left report “excellent salaries, comprehensive insurance, and respect,” while RN-2 stated, “Outside, they treat Lebanese nurses very well.” As a personal example, NS-4 shared that her sister became “supervisor of the department, being morally respected and well paid.” These shared success stories created a substantial 'pull' effect.

Factors Affecting Quality of Care

Nurses identified four major repercussions of migration that have negatively affected the quality of care, namely increased workload pressure, recruitment of inexperienced staff, psychological and emotional strain, and a decline in job satisfaction and motivation. Increased pressure due to workload is due to the severe nursing shortage that has dramatically increased the workload on remaining staff, leading to physical fatigue and reduced ability to meet patient needs. As RN-11 explained, “I used to have a maximum of five patients; now I am taking care of nine or 10 patients per shift…increasing my fatigue, as I can’t respond to all patients’ needs.” Another participant described a rise in complaints and miscommunication, as PN-4 noted, "Doctors' complaints increased for many reasons, as the current employees (new nurses) lack experience, in addition to the lack of coordination between the departments." The added responsibility of supervising new nurses also intensified the strain, as RN-2 shared, "More overtime, lots of supervision, more responsibility for new employees." Staff turnover contributes to workplace instability and decreases morale, as noted by RN-5: "Higher job turnover leads to discomfort for the employee, adding to increased doctors' and patients’ complaints." These challenges reflect a cyclical burden on the remaining workforce and the healthcare system at large.

Problems related to the recruitment of inexperienced nurses involve the migration of competent nurses, which has resulted in their replacement by inexperienced newly recruited staff, contributing to increased errors. One RN stressed, “New nurses often lack the skills and experience needed, which places additional stress on us and patient care” (RN-4). Broader workplace dissatisfaction followed: “Long work shifts, low salaries, lack of nurses, more patient complaints, and overtime all negatively affected nurse stability” (PN-5). Concerns about patient safety were frequently expressed, as RN-1 admitted, “Actually, I am living in constant fear of errors,” and PN-3 echoed, “With the newcomers, we are noticing new nursing errors in medications and other tasks or equipment use.” 

Aggravated psychological and emotional strain is the combined burden of long hours, reduced support, and a destabilized work environment that has taken a psychological toll. It was expressed by RN-7, who said, “I am tired psychologically. I am now more confused, and that led to a lack in my productivity to reply to patients’ calls,” and PN-4 declared, “All of this increased my fatigue, stress, and anxiety with more delays in responding to patient needs and complaints.” Similar experiences were echoed by RN-10, NS-1, and NS-4, who acknowledged that ongoing stress has undermined their ability to function effectively.

The decline in job satisfaction and motivation is caused by the ongoing pressure, financial hardship, and lack of support, which have led to diminished motivation and professional fulfillment. It was expressed by RN-9 that she was "very upset and even sad about my situation, and this dissatisfaction has completely decreased my enthusiasm for my work." Mandatory overtime and chronic stress worsened these issues, as RN-11 stated: "We worked overtime...sleep became less, anxiety increased a lot, with no efficient care delivered as patients are less satisfied." The absence of institutional support and growth opportunities further eroded morale, as RN-2 remarked, "I am no more motivated, as the quality of nursing care has decreased...zero experience."

Coping Strategies Adopted by Nurses

In response to ongoing crises, nurses adopted a variety of coping mechanisms to maintain personal and professional stability. These included seeking additional income, building resilience and adapting, and relying on family and social support. Seeking additional income was a necessity. Faced with high workloads, low pay, and a lack of institutional appreciation, many nurses sought alternative sources of income. Some nurses have increased their working hours within their institution; RN-4 shared, "I work extra time in extra departments," while others worked elsewhere. It was stated by RN-10 that, “I reduced my work inside the hospital and started working in six dispensaries; in addition, I am teaching in private technical institutions to raise my income.” Others secured contracts with international organizations, as NS-3 noted, “I work new overtime for fresh dollars with the World Health Organization for $25 per day.” Others turned to entrepreneurship: NS-3 explained, "I opened a new job … raising chickens and sheep … selling milk, meat, and eggs," while RN-7 shared, "I am working extra time with my husband in the cosmetic salon." Many also cut expenses: NS-1 noted, "I now select only the essential food to consume based on its cost and only buy fuel for my work," a stratagem echoed by others.

Building resilience and adapting was demonstrated by the nurses through professional commitment and personal adaptation. One nurse expressed, “The hospital appreciates me, so I will never give up on them” (RN-7). Many re-evaluated their priorities, as NS-3 shared, “Now I'm focusing more on things I feel more interested in, such as doing sports or drinking my coffee, or seeing a friend.” For others, the crisis brought unexpected benefits, such as family time despite the extra working hours: “I have dedicated much more time to my kids, which gave more meaning to my life,” noted RN-1. The crisis fostered greater coping capacities, as NS-6 reflected, “These crises made me more organized and decisive, as I can handle many tasks together, especially after my husband travelled.” Flexibility became key, as seen in NS-8’s approach: "I am working only three days physically and trying to reply to the team’s needs remotely." 

**Figure 1:**
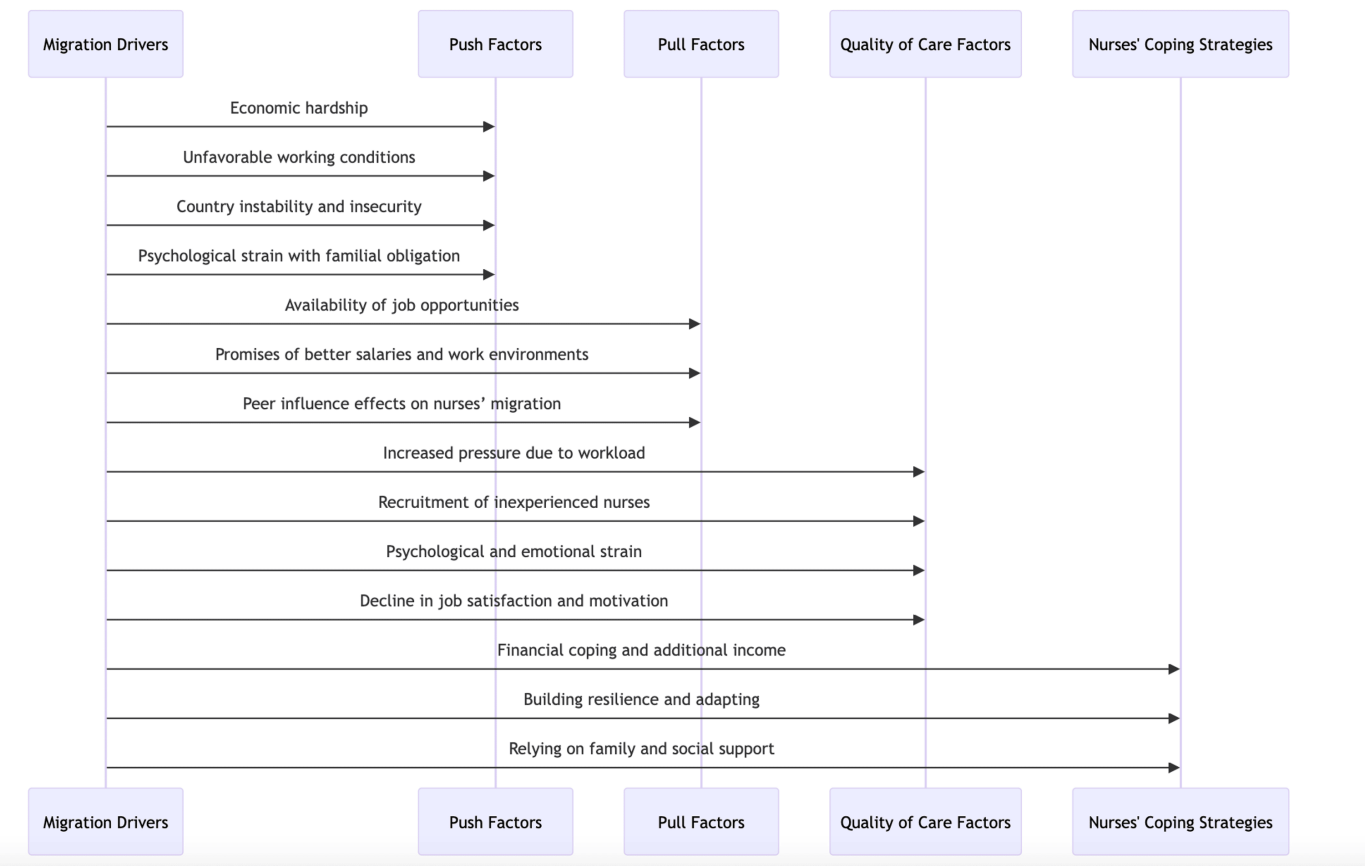
Diagram portraying push and pull dynamics of nurse migration and its implications for care quality and the resulting coping strategies identified in the study

Relying on family and social support has been crucial for the nurses. To navigate ongoing challenges, the nurse participants heavily relied on family bonds and social support systems, as RN-10 shared: "Building new relations with friends helped me in boosting my self-esteem." While RN-5 emphasized the role of family: "My parents were very supportive during this period; they welcomed my kids happily to give me much time to reorganize my life." This support extended further: NS-4 noted, “My children supported me with my husband; I found moral support that I did not expect.” Spouses also provided crucial psychological assistance, as RN-2 stated, “My husband encouraged me a lot to continue my education for a master’s degree.” Broader emotional support came from friends and colleagues; PN-4 said, “All were there for me; they valued me more, listened to my needs,” and PN-5 recalled, “I had one friend who stood with me in my difficult time, even in my household matters.” These findings are integrated into a conceptual model that represents the relationship between push and pull factors and their impact on care quality and coping mechanisms (Figure 1).

## Discussion

To the best of our knowledge, this is the first study to examine nurse migration drivers and consequences in Lebanon during multiple crises. Our findings uncover several new dynamics, including the reversal of family obligations as a migration driver, the intensification of economic hardship due to currency devaluation, the growing influence of peer networks on nurses’ decisions to migrate, the perceived decline in health care quality linked to migration repercussions, and creative coping strategies.

Economic hardship remains the primary push factor in our study, consistent with previous research in Lebanon and a systematic review of healthcare workers from LMICs, which showed that inadequate salaries and limited benefits drive nurse migration [9,17], while our findings also provide new insights specific to the current Lebanese context. However, the situation has intensified markedly due to the devaluation of the Lebanese currency, making current economic conditions far more severe. Poor working conditions, including high workloads, long hours, and job insecurity, also persist and align with global evidence linking unfavorable environments to burnout and intention to migrate [18, 19]. 

Further compounding the crisis, country instability and insecurity have deepened nurses’ sense of fear and hopelessness about the country's future. These findings reinforce previous research indicating that ongoing national unrest fuels the migration of skilled professionals, specifically Lebanese nurses from critical care units, emergency departments, and medical-surgical floors across various hospitals in Lebanon [20].

In addition, psychological strain and familial obligations were identified as key push factors driving nurses’ migration. Participants reported emotional distress from economic hardship and the pressure to support their families, viewing migration as essential for the well-being of their dependents, findings that are consistent with Konlan et al. and Labrague and de Los Santos [21,22]. Notably, our study reveals a shift in Lebanon: while earlier research found family ties encouraged nurses to stay [14,23], the current crisis has reversed this trend, with family responsibilities now driving migration in pursuit of better living conditions.

On the other hand, pull factors included the availability of attractive job opportunities abroad, especially in Gulf Cooperation Council (GCC) countries. These destinations offer attractive salaries, professional recognition, and secure employment, in stark contrast to Lebanon’s deteriorating job market. This pattern reflects global trends where high-income nations recruit nurses from economically distressed regions [14]. Another compelling pull factor included better salaries, improved work conditions, and opportunities for professional growth abroad. Participants highlighted the appeal of financial stability, professional respect, manageable workloads, and supportive work environments, reflecting motivational theories that value both monetary and non-monetary incentives [14]. Additionally, peer influence emerged as a new finding in the Lebanese context, with nurses describing colleagues’ positive migration experiences as validating their own decisions, echoing Afshari et al. [24] and underscoring the growing importance of social networks in shaping migration intentions.

Nurse migration has intensified workload pressures, leading to physical and emotional exhaustion, leading to reduced care quality, consistent with evidence linking poor staffing ratios to compromised healthcare outcomes [24]. A unique finding is the increased recruitment of inexperienced staff, raising concerns about patient safety and clinical competence. While noted globally [25], this issue is particularly critical in Lebanon due to the sharp loss of institutional expertise. Additionally, national instability and ongoing crises have amplified psychological strain among nurses, further impairing performance [24]. Emotional exhaustion, financial stress, and lack of administrative support emerged as key factors lowering job satisfaction and motivation, reflecting trends in Lebanon and other middle-income countries [26,27].

To cope with economic hardship, nurses adopted income-generating strategies such as extra shifts, additional jobs, and entrepreneurial activities, practices that, while necessary, may increase fatigue and impact care quality [28]. Complementary evidence from LMICs, including South Africa, also highlights dual practice and multiple jobholding among nurses, linking these coping strategies to tiredness, reduced alertness, and clinical risk [29], which further supports our interpretation. Despite challenges, many showed resilience and adaptability through self-care, professional development, and family engagement, reflecting behaviors linked to better mental health and reduced occupational stress [30]. However, resilience was notably lower among bedside nurses facing high workloads and limited support, a decline uniquely linked in this study to the compounded effects of national crises and institutional neglect [6]. Family and social support also played a critical role, offering emotional and practical relief. Strong ties with spouses, parents, and extended relatives enhanced nurses' ability to cope, in line with studies linking social support to healthier coping and higher retention [14,28,30]. These findings highlight the combined importance of economic, psychological, and relational strategies in helping nurses navigate prolonged crises.

Policy implications and strategic recommendations

This study highlights nurse migration as both a consequence and indicator of systemic weaknesses in Lebanon’s health system, driven by poor compensation, high workloads, and emotional exhaustion. A coordinated national response is urgently needed. At the national level, integrated workforce strategies should focus on retention, professional growth, and psychosocial support. Beyond salary reforms, policies must promote equitable resource allocation, inclusive workplaces, and resilience-building, such as mental health services, childcare access, flexible scheduling, and relocation support. Establishing a national health workforce observatory is essential for sustainable, data-driven planning.

At the regulatory and educational levels, nursing education should be aligned with real-world demands by incorporating leadership, resilience, and crisis management. The Order of Nurses should advocate for improved working conditions and mental health support. These recommendations offer a comprehensive framework to address nurse migration within a broader strategy for health system reform and workforce sustainability.

Limitations and strengths

While this qualitative study offers valuable insights into nurse migration in Lebanon, its findings are limited by the focus on hospital settings in North Lebanon and the absence of follow-up interviews, which may affect generalizability and depth. Nevertheless, it provides timely, context-specific evidence from a crisis-affected underserved region, amplifying the voices of nurses and offering a nuanced understanding of migration drivers, care quality, and coping strategies, insights that can inform future research and policy development.

## Conclusions

This study provides timely insights into nurse migration in North Lebanon amid overlapping national crises. While economic hardship, heavy workloads, and psychological strain remain central drivers, new dynamics have emerged. Family obligations, previously a reason to stay, now act as push factors due to the worsening of living conditions and financial collapse. Additional influences, such as peer encouragement and escalating national instability, further shape migration decisions.

The findings also highlight the impact of migration on healthcare, including the hiring of less experienced staff and a perceived decline in care quality. Although nurses employ coping strategies, their resilience is increasingly strained by institutional neglect and prolonged crisis exposure. These evolving patterns offer a deeper understanding of migration drivers within a complex and deteriorating system, distinguishing this study from prior research. Altogether, these findings offer valuable guidance for policymakers, healthcare leaders, and educators to strengthen nurse retention, improve care quality, and integrate resilience-building into workforce strategies in Lebanon.
